# Venereal Diseases at Guy's Hospital

**Published:** 1917-03-17

**Authors:** 


### VENEREAL DISEASES AT GUY'S HOSPITAL.

### The Working of the New Scheme.

It is less than a year since the publication of the final Report of the Royal Commission on Venereal Diseases, and that already a practical scheme should have been evolved adopting many of its recommendations is, taking into consideration the exceptional conditions now prevailing, a matter upon which the Public Health authorities deserve the warmest congratulation.

Those who read that Report will remember the numerous recommendations made for the prevention and improved treatment of these diseases, but in order to compare the new regulations with the Commissioners' findings the more important of them may be briefly summarised.

The need for accurate records in hospitals and Poor-Law establishments, and for the confidential registration of the causes of death, was especially emphasised. It was urged that every use should be made of the facilities offered by universities and hospitals for diagnosis by laboratory methods; the cost to be met as to 75 per cent. from Imperial funds and 25 per cent. from the local rates.

Measures should be taken to render the best modern treatment of venereal diseases readily available for all classes of the community under conditions of secrecy, and special clinics provided for the working classes at suitable hours. Salvarsan or its substitutes should be supplied gratuitously, subject to proper safeguards. Medical students and practitioners should have access for educational purposes to any institution dealing with these diseases as part of a local authority's scheme.

Cards of warning should be issued by doctors to their patients informing them of the contagious nature of the disease they have contracted.

The advertisement of any patent remedy for the cure of these diseases should be made a criminal offence. Whether by a compulsory course of instruction or otherwise, steps should be taken to ensure that every medical student has an adequate knowledge of these diseases and questions set on them in examination papers. Infectious venereal disease should constitute an incapacity for marriage, and any communication made by a medical man to the parent or guardian of a man or woman about to contract such a marriage should be regarded as privileged.

The Commissioners further emphasised the intimate correlation between alcoholism and overcrowded, insanitary dwellings and the contraction and spread of these diseases. They expressed themselves, moreover, as "deeply sensible of the need and importance of the appeals to conscience and honour made by religious bodies and associations formed for this purpose."

It is evident, therefore, that these regulations deal—as, indeed, they could only be expected to deal—with a limited section of the recommendations. Other and equally important measures of reform have been introduced in the Home Secretary's Bill, now before the House of Commons, and it seems as if at last the State is determined to gain the upper hand of this scourge of our national life.

This scheme may be appropriately divided into two sections: (1) the provision of extended facilities for general practitioners to acquire knowledge of modern methods of diagnosis and intravenous medication; and (2) the establishment of venereal clinics at a number of institutional centres.

All the practitioners in the London postal area have been circularised, the scheme being fully explained to them, together with a list of institutions from which they can obtain the necessary material for obtaining blood for the Wassermann examination and specimens for cultural purposes, and to which they can send their patients for diagnosis or treatment. They have also been provided with specimen forms of warning to be issued to those suffering from gonorrhœa and syphilis respectively, or who have had a dose of salvarsan. Arrangements are, it is understood, in progress for the delivery of a course of lectures at certain approved centres, and qualified practitioners will be invited to take posts in these as clinical assistants, at rates of remuneration fixed by the Local Government Board. Salvarsan will only be issued to those who have produced satisfactory evidence that they are practised in its administration. As regards institutional treatment, arrangements have been entered into at twenty-two hospitals for the free treatment of venereal patients on three or more days per week.

The times are, in the main, such as will not interfere with the occupation of the artisan classes. Thus nearly all have male clinics on one or two days a week at 5.30 P.M. or later, and for women at 10 A.M. or in the early afternoon. The centres are well distributed, and all the big teaching hospitals are represented, with the exception of St. Bartholomew's.

To judge of the practical working of the scheme a typical centre—that at Guy's Hospital—may be briefly described. For some years past the excellent genito-urinary clinic has been under the charge of Mr. A. R. Thompson, F.R.C.S., with two out-patient days a week; cases suitable for vaccine treatment and microscopic material have been sent to Dr. Eyre, the head of the bacteriological department. These departments have now both been extended, and the days of attendance for males and females increased from two to four. Two assistants—Messrs. Gathergood and Andrews—have been appointed to deal with the men patients in a conveniently isolated set of rooms—originally designed to deal with phthisis patients—on the first floor at the back of the out-patient hall. Here there is ample room to examine each patient in privacy, and a well-equipped laboratory adjoining enables specimens to be examined for spirochætes by dark ground illumination and pus for gonococci with a minimum of delay. To the women's and children's department a lady—Miss Rawlings, M.B. Lond.—has been appointed, and these clinics are held at noon.

An additional assistant works under Dr. Eyre in the bacteriological department—Miss Griffin, M.B., D.P.H. The departments are under the charge of the genito-urinary surgeon, the bacteriologist, and, in the case of the women's side, of the obstetric surgeons.

There are three beds available for each sex, to which cases needing salvarsan are admitted, and the cases are changed three times weekly. On the women's side neo-salvarsan is given to out-patients, who merely rest a few hours after the administration. This, we understand, is also to be introduced in the men's department, thus making a far greater number of treatments possible. Patients can be sent on to this clinic by any of the physicians or surgeons to whose out-patient consultations they come, by the obstetric surgeons and the dermatologist; many attend direct. In the first month (January 1917) sixty men were treated and fifty-six women, and fifty samples of blood were examined. The system of registering attendance is extremely simple, and the names and addresses of the patients are kept in a confidential book to which the physician treating the case alone has access. In all clerical work they are designated by numbers only.

It is hoped shortly to arrange for three-month courses of lectures and practical demonstrations, at which all medical men will be free to attend, and these will be held three times per annum. Already there has been a series of lectures delivered by Messrs. Thompson and Eyre on the treatment and bacteriology of venereal diseases, which were fairly well attended by both students and outside practitioners. Though obviously in working far too short a time for a fair estimate to be made of its chances of success in reaching the class of case hitherto neglected or treated by quacks, the number of cases treated in the first month certainly gives grounds for such a hope.

